# A neglected risk for sparganosis: eating live tadpoles in central China

**DOI:** 10.1186/s40249-017-0265-7

**Published:** 2017-05-04

**Authors:** Jing Cui, Ye Wang, Xi Zhang, Xi-Meng Lin, Hong-Wei Zhang, Zhong-Quan Wang, Jia-Xu Chen

**Affiliations:** 1 Department of Parasitology, Medical College, Zhengzhou University, Zhengzhou, China; 2 Department of Emergency, Peking University Third Hospital, Beijing, China; 3 Institute of Parasitic Diseases, Center for Disease Prevention and Control of Henan Province, Zhengzhou, China; 4 National Institute of Parasitic Diseases, Chinese Center for Disease Control and Prevention; Key Laboratory of Parasite and Vector Biology, Ministry of Health, Shanghai, China

**Keywords:** Sparganosis, Sparganum, Tadpole, Central China

## Abstract

A 29-year-old farmer from central China was sent into the Emergency Department of the Affiliated Hospital of Zhengzhou University. He had a 15-day history of persistent high fever, abdominal distention and pain. The patient was clinically diagnosed as appendicitis and peritonitis, and treated with antibiotics in a local hospital, did not improve. On exploratory laparotomy, the appendicular perforation and peritonitis were seen; appendicectomy were performed, and antibiotics were given. However, high fever and abdominal pain still persisted; intestinal adhesion and obstruction, ascites appeared. He was given the “critically ill notice”. He had eosinophilia (12.95%) and the history of eating live frog tadpoles for treating his cutaneous pruritus 3 days before onset of the disease. Serum anti-sparganum antibodies assayed by ELISA were positive. This patient has hospitalized for one and half months and spend more than US$ 12 000. This patient was primarily diagnosed as visceral sparganosis, and cured with praziquantel.

Sparganosis is one neglected but important parasitic zoonosis of poverty. Human infections were mainly acquired by eating raw or uncooked meat of frogs and snakes infected with plerocercoids, using frog or snake flesh as poultices, or drinking raw water contaminated with infected copepods. However, sparganosis caused by ingestion of live tadpoles are emerging in central China. Our surveys showed that 11.93% of tadpoles in Henan province are infected with plerocercoids. Eating live tadpoles is a high risk for sparganum infection. The comprehensive public health education should be carried out for people in endemic areas and the bad habit of eating live tadpoles must be discouraged.

## Background

On 20 August last year, a 29-year-old farmer from Qixian County of Henan Province, central China, was sent into the Emergency Department of the Affiliated Hospital of Zhengzhou University. He had a 15-day history of persistent high fever, abdominal distention and pain. The patient was clinically diagnosed as appendicitis and peritonitis, and treated with antibiotics in a local hospital, did not improve. On exploratory laparotomy, the appendicular perforation and peritonitis were seen; appendectomy was performed, and antibiotics were given. However, high fever (39–41 °C) and abdominal pain still persisted; the intestinal adhesion and obstruction appeared after operation. His blood routine examination revealed a normal white blood cell count of 5.33 × 10^9^/L (normal range, 3.5–9.5 × 10^9^/L), but with eosinophilia (12.95%, 0.69 × 10^9^/L; normal range, 0.02–0.45 × 10^9^/L, 0.4–0.8%). The rest was as follows: red blood cells, 2.93 × 10^9^/L (4.3–5.8 × 10^9^/L); hemoglobin, 88 g/L (130–175 g/L); C-reactive protein, 53.5 mg/L (1–10 mg/L). No bacteria were found on blood culture, all the IgM antibodies to virus, mycoplasma and chlamydia were negative. Peritoneal, pelvic, and bilateral pleural effusion were seen on CT image, examination of peritoneal and pleural effusion showed leukocytosis of 9 707/L (normal range, 0–100 × 10^6^/L). For his situation, he was given the “critically ill notice”.

On further carefully inquiring, our patient had the history of eating live frog tadpoles (80 tadpoles in 2 doses for 5 days) for treating his cutaneous pruritus 3 days before onset of the disease. Thus, parasitic diseases were highly suspected on this history, together with clinical manifestation and eosinophilia. The serum specific antibody IgG against tissue-dwelling parasites (*Clonorchis sinensis*, *Paragonimus skrjabini*, *Schistosoma japonicum*, metacestode of *Taenia solium*, and *Trichinella spiralis*) were assayed by ELISA or immunofluorescence test, and negative. Serum samples were also assayed by ELISA using *S. erinaceieuropaei* sparganum excretory–secretory (ES) antigens and recombinant sparganum cysteine protease as antigen [1, 2], and specific anti-sparganum antibody IgG was positive. By this time, the patient has hospitalized for one and half months and spend more than US$ 12 000 (including the expense for medical examination, medicine, operation, hospitalization and so on). This patient was preliminary diagnosed as visceral sparganosis and treated with praziquantel (75 mg/kg in 3 doses per day for 3 days). Three days after treatment, there was general improvement with fever declining to 37.5 °C. After treatment with two courses of praziquantel, fever and abdominal pain disappeared, but he also had eosinophilia (10.1%, 0.70 × 10^9^/L) and positive anti-sparganum antibodies.

## Discussion

### Common mode of sparganum infection in humans

Sparganosis is a neglected parasitic zoonosis resulted from infection with the plerocercoid larvae (spargana) of some diphyllobothroid tapeworms belonging to the genus *Spirometra*. The most important species of the genus *Spirometra* with plerocercoids that cause human sparganosis include *Spirometra erinaceieuropaei* (syn. *S. erinacei* or *S. mansoni*) and *S. mansonoides. S. erinaceieuropaei* is most frequently found in Asia and Africa, whereas *S. mansonoides* is mainly found in North America [3, 4]. Adults parasitize in small intestine of dogs, cats, and other mammals. The first intermediate hosts are freshwater copepods (cyclops), whereas the second intermediate or paratenic hosts include amphibians and reptiles (e.g., frogs, snakes and pigs) [5]. Human are accidental hosts. Human become infected mainly by eating raw or undercooked meat of frogs and snakes infected with plerocercoids, using frog or snake flesh as poultices for treatment of skin diseases or eye inflammations, or drinking raw water contaminated with infected copepods [6, 7]. So, sparganosis is not only a food-borne zoonotic disease, but also a water-borne and contact-transmitted zoonosis. Once ingested, the larvae penetrate the gut wall and enter into peritoneal cavity; then these larvae may invade the muscle or subcutaneous tissues, and other organs. Plerocercoids are unable to penetrate intact skin, but they can penetrate through skin lesions, eyes, mucosa, etc.

### Eating live tadpoles is another mode of sparganum infection

In China, more than 1 300 cases with sparganosis were reported and distributed in 29 out of 34 Provinces/Autonomous Regions/Municipals during 1949–2014 [8]. Most of cases with sparganosis were found in southern China and were caused by eating raw or undercooked frog or snake meat, or using raw meat as poultices. Sparganosis is rarely seen in central and northern China. However, since 2006, more than 30 autochthonous cases caused by ingestion of live frog tadpoles have emerged in Henan province of central China [9]. The inhabitants in Henan did not have the traditional habit of eating raw flesh and tadpoles, and using raw flesh as poultices. But, in recent years, eating live tadpoles have become a self-treatment method in some rural areas of Henan; some villagers believe that tadpoles to have the medicinal effects for treating skin diseases (psoriasis, scabies, eczema and cutaneous pruritus), so educate their children to eat live tadpoles. Our survey showed that 11.93% (163/1 366) of tadpoles and 16.23% (565/3 482) of frogs (*Rana nigromaculata, R. limmochari, and R. temporaria*) in Henan province are infected with plerocercoids, and all the plerocercoid isolates from Henan were identified as *S. erinaceieuropaei* [10, 11]. An epidemiological study showed that 56.71% (169/298) of inhabitants in one village of Henan had the history of eating live tadpoles, and the prevalence of serum anti-sparganum IgG in inhabitants who had the history of eating tadpoles (9.47%) was obviously higher than those who did not (0.78%) (*P* < 0.01) [12]. This patient reported here got sparganosis by swallowing live tadpoles.

### Clinical presentations of sparganosis

After entry to humans, the plerocercoids migrate and locate in different parts of the body (such as subcutaneous tissues, muscles, eyes, mouth, brain, breasts, lungs, abdominal organs, urogenital tract pleura, pericardium, and spinal canal) [3]. Clinical presentations depend on the number and location of the larvae. The early stages of human sparganosis may be asymptomatic, but the migrations and secretions of living plerocercoids often cause the localized inflammatory reaction and edema of the surrounding tissues. The most common clinical manifestation of sparganosis is the migratory subcutaneous nodules that may appear and disappear over a period of time. The nodules usually itch, swell, turn red, and are often accompanied by painful edema. Chill and fever may accompany this infection [8]. Eye infection which is particularly common in Asia produces conjunctivitis and swelling [5]. Headaches, seizures, hemiparesis are common symptoms of cerebral sparganosis. Eosinophilia is a common sign in patients with spargarnosis [13, 14].

### Clinical features of sparganosis caused by eating live tadpoles

The clinical manifestations of sparganosis caused by eating live tadpoles were obviously different from that resulted from eating raw or uncooked meat, or using flesh as poultices in southern China and other countries, where the patients usually had only one local lesion (mainly the ocular, oral or subcutaneous lesions); whereas the patients having history of eating live tadpoles usually had general presentations and multiple lesions, the prominent typical manifestations were persistent high fever, abdominal pain, eruption, and eosinophilia; peritonitis, intestinal adhesion or obstruction, abdominal or pleural effusion occurred sometimes as above-mentioned in our patient. The different manifestations may be related to the mode and severity of infection. The plerocercoid (1–13 cm long and 1–2.5 mm wide) in frog or snake flesh is large enough to be often seen by naked eye; the larvae can be picked up and discarded during processing of flesh or before using as poultices. So, the patients who had the history of eating raw or semi-cooked meat or used raw meat as poultices, usually get the light infection, and have the local manifestations [5, 8]; whereas the patients who had the history of eating live tadpoles often get the severe infection and have general manifestations [15].

### Diagnosis and differential diagnosis of sparganosis

Because sparganosis is a rare disease in northern and central regions of China, it is easily neglected and previously most of cases might be undiagnosed or misdiagnosed. This disease is not only a public health hazard but also produce a major medical expenses.

The clinical diagnosis of human sparganosis is rather difficult because the symptomatology is not pathognomonic. A biopsy of subcutaneous nodule requires the intervention of a surgeon and the definite diagnosis of sparganosis can be made by detection of the larvae in a biopsy specimen, but the confirmative diagnosis is very difficult for visceral and cerebral sparganosis since the larva is found only by surgical removal. Hence, serological tests are the most common used diagnostic methods for sparganosis. The ELISA using *S. erinaceieuropaei* sparganum ES antigens for detection of specific anti-sparganum antibodies had a good sensitivity and specificity, but it still has cross-reactivity with sera of patients with cysticercosis and paragonimiasis [1]. The ELISA using recombinant or native sparganum cysteine protease as antigens has a higher sensitivity (100%) and specificity (97–98.22%) for serodiagnosis of human sparganosis, compared with ELISA using sparganum ES antigens. [2, 16].

Visceral sparganosis with fever, abdominal pain and eosinophilia should be differentiated from acute clonorchiasis, paragonimiasis, schistosomiasis, and gnathostomiasis. Serological tests and the history of consuming frog or snake meat, using raw meat as poultices, eating live tadpoles, or the ingestion of contaminated water are the keys to differential diagnosis of the disease, as described previously in this patient.

### Treatment and control of sparganosis

Surgical removal of the larvae is the most effective therapy for subcutaneous, ocular, oral, and cerebral sparganosis. The whole worm body should be completely removed; because a remaining scolex will lead to recurrence of this disease. Praziquantel is the drug of first choice for multiple subcutaneous sparganosis and visceral sparganosis. The recommended total dose is 120–300 mg/kg divided into two or three dose per day for 2–3 days [17, 18], but praziquantel has no obvious therapeutic efficacy for cerebral sparganosis [19]. Our patient was cured with two courses of praziquantel, but the eosinophilia and serum anti-sparganum antibody level decline slowly after treatment.

## Conclusion

In endemic areas of sparganosis, people should be advised of the dangers of eating raw or undercooked frog or snake meat, using raw meat as poultices or drinking untreated water. Water from ponds and ditches should be boiled or filtered before drinking. Moreover, eating live tadpoles is a high risk behavior for sparganum infection. The local governments, public health officials and medical practitioners should be aware of such risks and implement strategies to eliminate them. The best ways to prevent human sparganum infections are to carry out the comprehensive public health education and to discourage the bad habit of eating raw meat and live tadpoles, using raw meat as poultices or drinking untreated water for people in endemic areas.

## Abbreviations

ELISA: Enzyme-linked immunosorbent assay
ES: Excretory–secretory
